# The landscape of PBMCs in AQP4‐IgG seropositive NMOSD and MOGAD, assessed by high dimensional mass cytometry

**DOI:** 10.1111/cns.14608

**Published:** 2024-02-09

**Authors:** Mengyuan Yao, Wenjing Wang, Jiali Sun, Tianshu Guo, Jiangping Bian, Fuyao Xiao, Yuanyuan Li, Hengri Cong, Yuzhen Wei, Xinghu Zhang, Jianghong Liu, Linlin Yin

**Affiliations:** ^1^ Department of Neuroinfection and Neuroimmunology, Beijing Tiantan Hospital Capital Medical University Beijing China; ^2^ China National Clinical Research Center for Neurological Diseases, Beijing Tiantan Hospital Capital Medical University Beijing China; ^3^ Beijing Institute of Hepatology, Beijing Youan Hospital Capital Medical University Beijing China; ^4^ Department of Neurology, Xuanwu Hospital Capital Medical University Beijing China; ^5^ Collaborative Innovation Center for Brain Disorders, Beijing Institute of Brain Disorders Capital Medical University Beijing China

**Keywords:** aquaporin‐4‐IgG seropositive NMOSD, mass cytometry, MOGAD, PBMCs

## Abstract

**Objectives:**

Data on peripheral blood mononuclear cells (PBMCs) characteristics of aquaporin‐4 (AQP4)‐IgG seropositive neuromyelitis optica spectrum disorder (NMOSD) and myelin oligodendrocyte glycoprotein antibody‐associated disease (MOGAD) are lacking. In this study, we describe the whole PBMCs landscape of the above diseases using cytometry by time‐of‐flight mass spectrometry (CyTOF).

**Methods:**

The immune cell populations were phenotyped and clustered using CyTOF isolated from 27 AQP4‐IgG seropositive NMOSD, 11 MOGAD patients, and 15 healthy individuals. RNA sequencing was employed to identify critical genes. Fluorescence cytometry and qPCR analysis were applied to further validate the algorithm‐based results that were obtained.

**Results:**

We identified an increased population of CD11b+ mononuclear phagocytes (MNPs) in patients with high expression of CCR2, whose abundance may correlate with brain inflammatory infiltration. Using fluorescence cytometry, we confirmed the CCR2+ monocyte subsets in a second cohort of patients. Moreover, there was a wavering of B, CD4+ T, and NKT cells between AQP4‐IgG seropositive NMOSD and MOGAD.

**Conclusions:**

Our findings describe the whole landscape of PBMCs in two similar demyelinated diseases and suggest that, besides MNPs, T, NK and B, cells were all involved in the pathogenesis. The identified cell population may be used as a predictor for monitoring disease development or treatment responses.

## Graphical abstract

Our data systematically characterized the circulating PBMCs landscape of AQP4‐IgG seropositive NMOSD and MOGAD patients, revealed the profiles of periphery blood immune cells which may play potential roles in disease development or protection.

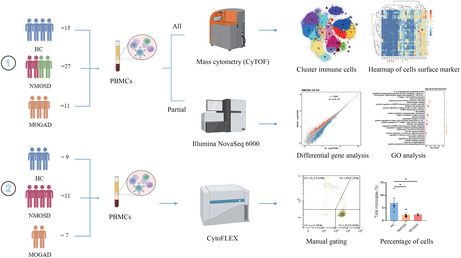

## 1 INTRODUCTION

Neuromyelitis optica spectrum disorder (NMOSD) is a recurrent inflammatory central nervous system (CNS) disease that predominantly impacts the optic nerves and spinal cord. Antibodies against the astrocyte‐expressed water channel aquaporin‐4 (AQP4)‐IgG are present in about 80% of patients. However, serum antibodies against myelin oligodendrocyte glycoprotein (MOG‐IgG) may be detected in AQP4‐IgG seronegative NMOSD patients. MOG‐IgG‐associated disease (MOGAD) has been deemed a distinctive CNS demyelinating disease that primarily manifests as optic neuritis, transverse myelitis and acute disseminated encephalomyelitis. Patients with AQP4‐IgG seropositive NMOSD or MOGAD frequently have analogous clinical and radiographic characteristics. Despite different studies performed to discriminate against them in recent years, numerous questions are still unresolved.^1^, ^2^, ^3^

To deeply comprehend the pathogenesis of AQP4‐IgG seropositive NMOSD and MOGAD, a powerful approach cytometry by time‐of‐flight mass spectrometry (CyTOF) to map and phenotypes of circulating immune cell subsets in peripheral blood mononuclear cells (PBMCs) is needed.^4^, ^5^ Here, we use a panel of 36 metal isotope‐conjugated antibodies that target intrinsic or cell surface markers presented inside or on PBMCs and describe their phenotypes among AQP4‐IgG seropositive NMOSD, MOGAD patients, and healthy controls (HCs). Afterward, we were able to confirm the different populations of different monocyte subclasses by fluorescence cytometry, screen pivotal candidate genes by RNA sequencing, and verify their expressions by qPCR analysis. Our results unveil distinct signatures of PBMCs between patients with AQP4‐IgG seropositive NMOSD and MOGAD and provide novel directions for therapeutic intervention for these two inflammatory demyelinated disorders.

## 2 METHODS AND MATERIALS

### 2.1 Patients and controls

We recruited blood specimens from 27 patients with AQP4‐IgG seropositive NMOSD (12 in acute and 15 in remission), 11 patients with MOGAD (all in acute), and 15 HCs for CyTOF analysis. The major clinical features of people who joined in mass cytometry were listed in Table 1, and further detailed information of patients was shown in Table S1. We recruited 11 patients with AQP4‐IgG seropositive NMOSD, 7 MOGAD patients, and 9 HCs for the flow cytometry confirmation analysis. All patients were in the acute phase. The major clinical features of people who joined in fluorescence cytometry were listed in Table 2. All NMOSD patients met the 2015 NMOSD diagnostic criteria, and MOGAD fulfilled the 2018 diagnostic criteria, and the 2023 international MOGAD panel proposed criteria.^6^, ^7^, ^8^ All blood samples were obtained from patients of the Department of Neuroinfection and Neuroimmunology, Beijing Tiantan Hospital, affiliated of Capital Medical University, from Jan 2021 to Nov 2023. AQP4‐IgG and MOG‐IgG titers were measured by cell‐based assay (CBA). Patients who experienced exacerbations or symptom onset within 2 weeks were recruited as acute phase; patients who had remained relapse‐free for at least 3 months were recruited as remission phase. All patients had no other autoimmune comorbidities or infectious diseases when the blood was being drawn. The study was approved by Beijing Tiantan Hospital's Ethics Committee (No. KY2019‐050‐02), and each participant provided informed consent in writing.

**TABLE 1:** Demographic and clinical characteristics of patients who participated in mass cytometry.

	NMOSD		MOGAD	HC	*p*
No. of patients (*n*=)	Acute (*n* = 12)	Remission (*n* = 15)	*n* = 11	*n* = 15	
Gender (F/M)	9/3	15/0	4/7	12/3	0.04^a^
Age (years), mean (SD)	46.3 (14.3)	42.5 (14.2)	35.0 (17.1)	45.4 (7.6)	
EDSS, median (range)	4.3 (2.5–8.0)	2.5 (0.0–7.0)	3.0 (0.0–5.0)	–	0.02^c^
Serum antibody titer median (range)	1:32 (1:10–1:320)	1:32 (1:10–1:320)	1:32 (1:10–1:100)	–	
QAlb (×10^−3^), median (range)	6.4 (2.7–10.8)	–	7.4 (5.8–12.3)	–	
IgG index, (range)	0.5 (0.4–1.2)	–	0.5 (0.4–0.7)	–	
Time since last attack (months), median (range)	5 (1–7)	6 (1–48)	18 (6–84)	–	0.03^b^

Abbreviations: EDSS, Expanded Disability Status Scale; QAlb, albumin quotient, presented as CSF‐to‐serum albumin ratio.

^a^ MOGAD versus HC.

^b^ NMOSD‐acute versus MOGAD.

^c^ NMOSD‐acute versus NMOSD‐remission.

**TABLE 2:** Demographic and clinical characteristics of patients who participated in spectral flow cytometry.

	NMOSD	MOGAD	HC	*p*
No. of patients (*n* =)	*n* = 11	*n* = 7	*n* = 9	
Gender (F/M)	10/1	6/1	8/1	
Age (years), mean (SD)	52.9 (12.4)	28.9 (9.6)	45.0 (12.0)	0.01^a^
EDSS, median (range)	5.5 (2.5–7.5)	2.0 (0.0–6.5)	–	0.02^b^
Serum antibody titer median (range)	1:100 (1:10–1:320)	1:100 (1:10–1:320)	–	
QAlb (×10^−3^) median (range)	5.6 (1.2–9.9)	5.8 (4.5–7.5)	–	
IgG index (range)	0.6 (0.5–0.6)	0.5 (0.5–0.7)	–	
Time since last attack (months), median (range)	5.5 (3.0–6.0)	6.3 (5.5–7.0)	–	

Abbreviations: EDSS, Expanded Disability Status Scale; QAlb, albumin quotient, presented as CSF‐to‐serum albumin ratio.

^a^ MOGAD versus HC.

^b^ NMOSD versus MOGAD.

### 2.2 Blood sample gathering and PBMCs separation

Samples of the patients who fulfilled the AQP4‐IgG seropositive NMOSD or MOGAD diagnostic criteria during the acute phase were collected before the administration of any immunotherapy. Fresh whole blood samples (6 mL) were drawn into anticoagulant tubes containing ethylenediaminetetraacetic acid (EDTA), and the plasma was removed by centrifuging the tubes. PBMCs were immediately isolated using SepMate tubes containing Ficoll and placed in liquid nitrogen using 90% FBS and 10% DMSO. Some PBMCs were divided into two sections; one was immersed again in a staining solution for CyTOF analysis, and the other was combined with a TRIzol reagent for RNA sequencing. The usage of each sample from different patients is shown in Table S1.

### 2.3 Mass cytometry

Frozen PBMCs were quickly thawed in a water bath at 37°C. and then were twice washed in RPMI 1640 supplemented with 10% FBS, penicillin‐streptomycin, and 10 U/mL Benzonase. The samples were then immersed again in PBS and stored on ice for subsequent application. PBMCs viability was typically >95% (Figure S1).

Before CyTOF, the antibody inside the cell or on the cell surface was labeled with the optimal concentration of metal‐conjugated antibody, and the cell concentration was adjusted to meet the detection requirements.^9^, ^10^ To identify a wide variety of immunocytes, a panel of 36 antibodies was performed. The reporter isotopes and antibodies are shown in Table S2.

### 2.4 CyTOF analysis

Data were acquired as FCS files, and analysis of the data was carried out in accordance with earlier research.^10^ Cytobank (www.cytobank.org) was used for analyzing CyTOF data. Table S3 displayed the identified cell types. As previously reported, the cell types were marked by manual gating.^11^ ViSNE algorithms were applied on the directed gated cells.^12^ The hierarchical analysis of cluster was conducted using the automated clustering gate capabilities. Heatmaps were made with R program (version 4.2.2).

### 2.5 Flow cytometric analysis

PBMCs were treated with Human TruStain FcX™ and maintained for 6 min at 25°C. Samples were tagged with a mixture of several monoclonal antibodies for 15 min at 4–8°C in the dark. CytoFLEX demonstrated surface protein expression. CytExpert software 2.3 was utilized to calculate the average fluorescence intensity. The antibodies selected are shown in Table S4.

### 2.6 RNA sequencing

An analysis of PBMCs from 6 AQP4‐IgG seropositive NMOSD and 3 MOGAD patients compared to 3 HCs was conducted using RNA sequencing (RNA‐seq). Briefly, mRNA was enriched by poly‐A pull‐down, followed by library preparation, verified by Agilent Bioanalyzer, sample purity and size observed at 260 bp, and finally sequenced by Illumina NovaSeq 6000. The above procedures were carried out at SinoTech Genomics Co., Ltd.

### 2.7 qPCR identification

The TRIzol™ reagent extracted total RNA from PBMCs as previously described.^13^, ^14^ A NanoDrop® ND‐1000 spectrophotometer was utilized to ascertain the concentration and purity of RNA. Making use of random hexamer primers, the Transcriptor First Strand cDNA Synthesis Kit, and anchored oligo (dT), RNA was utilized to create cDNA. CCR2 mRNA expression was compared to glyceraldehyde‐3‐phosphate dehydrogenase using SYBR Green‐based qPCR. All primer sequences were listed in Table S5.

### 2.8 Data analysis

For all statistical studies, GraphPad Prism 8.0 and IBM SPSS Statistics 20.0 were utilized. To determine if data had a normal distribution, the Shapiro–Wilk test was utilized. Data that adhered to a normal distribution were analyzed using the 2‐tailed unpaired Student's *t*‐test; data that did not were analyzed using the Mann–Whitney *U* test. The count data was compared using the chi‐squared test. All of the data were displayed using the mean ± SD or standard error of the mean (SEM). For *p* values less than 0.05, the statistical significance threshold was assumed.

## 3 RESULTS

### 3.1 Identification of PBMCs phenotypes among AQP4‐IgG seropositive NMOSD, MOGAD, and HC groups

**FIGURE 1:**
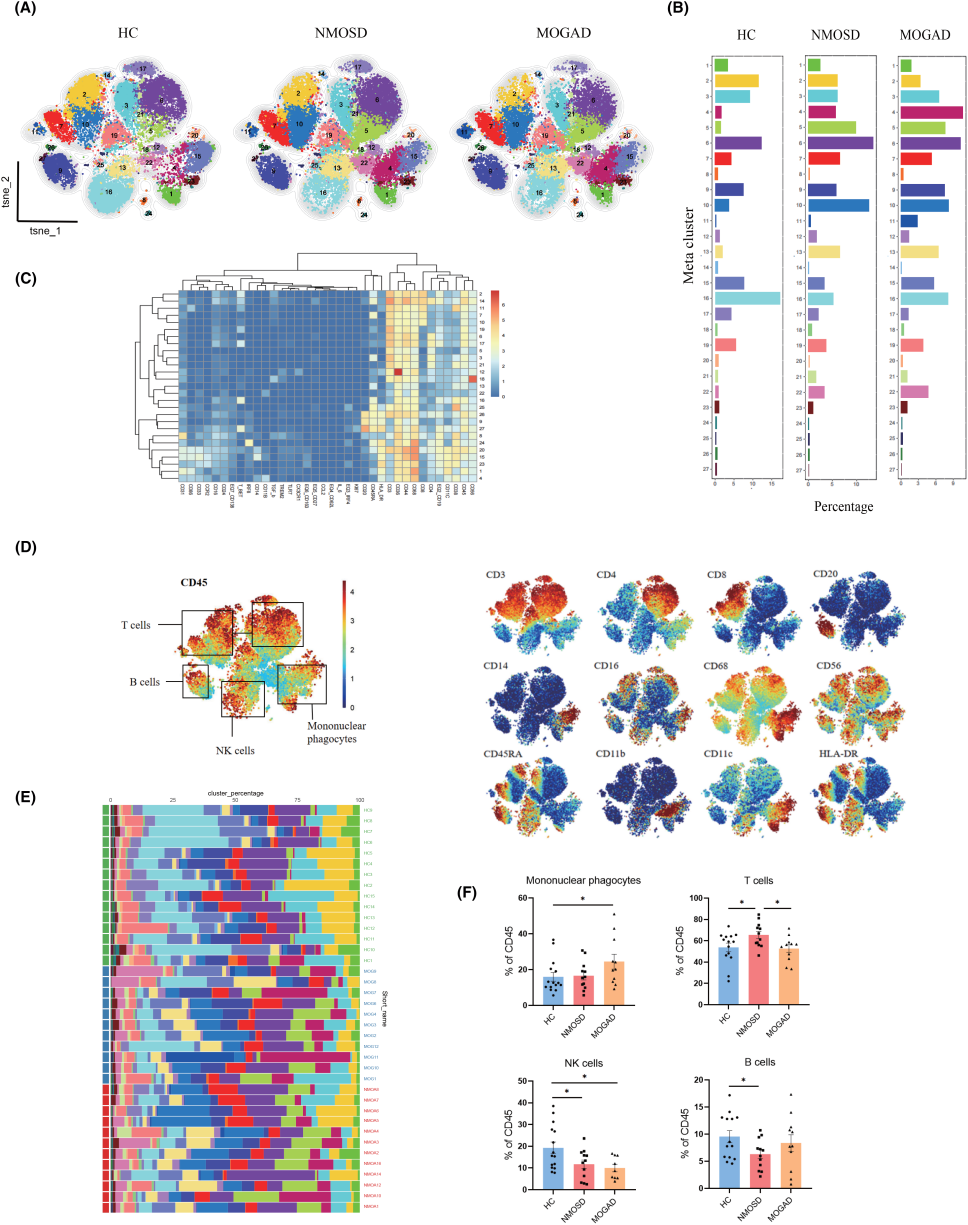
Single‐cell mass cytometry profiling of PBMCs in healthy controls (HC), or patients with AQP4‐IgG seropositive NMOSD and MOGAD during acute phase. (A) According to the relative expression markers, high dimensional mass cytometry clustering reveals distinct cell type subpopulations. CD45+ PBMCs were divided into 27 clusters. About 4000 CD45+ PBMCs per sample were included in the viSNE analysis. (B) Cluster abundances in HC and patients with AQP4‐IgG seropositive NMOSD or MOGAD. (C) A heatmap shows the relative expression levels of selected markers in the 27 t‐SNE clusters. (D) ViSNE plots of complete immune systems according to the relative expression of CyTOF markers in all samples. The cell populations are also indicated (left). (E) A histogram shows the cluster percentage of all participants in the 27 t‐SNE clusters. (F) Column graphs showing the frequencies for each subset of CD45+ PBMCs in HC, NMOSD and MOGAD groups (HC = 15, NMOSD = 12, MOGAD = 11). Data were presented as mean ± SEM; bars represent maximum and minimum values, and dots represent individual samples of each group. *p < 0.05.

CyTOF analysis was used to compare the characteristics of immune cell subsets at single‐cell levels of PBMCs in patients with NMOSD or MOGAD (both in acute phases) to those in HCs. The original gating mechanisms utilized for CD45+ cell identification are shown in Figure S1. We identified 27 clusters, which were superimposed on the t‐distributed stochastic neighbor embedding (t‐SNE) map in Figure 1A. The proportion of each cluster is depicted in Figure 1B. The expression profiles of each cluster were presented in a heatmap (Figure 1C). Variations in the immune cell population abundances were revealed by the viSNE map of obtained CD45+ PBMCs. ViSNE was applied to map all samples, and spectrum maps were utilized to show surface marker expression (Figure 1D). The cluster percentage of all individuals was displayed in a histogram (Figure 1E). Furthermore, we compared the percentages of MNPs, T, NK, and B cells among the three studied groups (Figure 1F). The amount of MNPs was considerably higher in the MOGAD group compared to HC group, while no obvious differences were discovered between NMOSD and HC groups. The amount of T cells was considerably increased in AQP4‐IgG seropositive NMOSD compared to both MOGAD and HC groups. Compared to HC group, the box charts showed a marked decrease in NK cells in both NMOSD and MOGAD patients. Unexpectedly, during the acute phase, the percentages of B cells decreased significantly in samples of AQP‐IgG seropositive NMOSD patients, compared to HC. The percentages of T cells in AQP4‐IgG seropositive NMOSD and MOGAD patients differed statistically (Figure 1F), indicating that the two demyelinated illnesses have different pathogenesis.

### 3.2 Lower CD14+ and higher CD11b+ MNPs in the proportion of NMOSD and MOGAD patients compared to HC group

**FIGURE 2:**
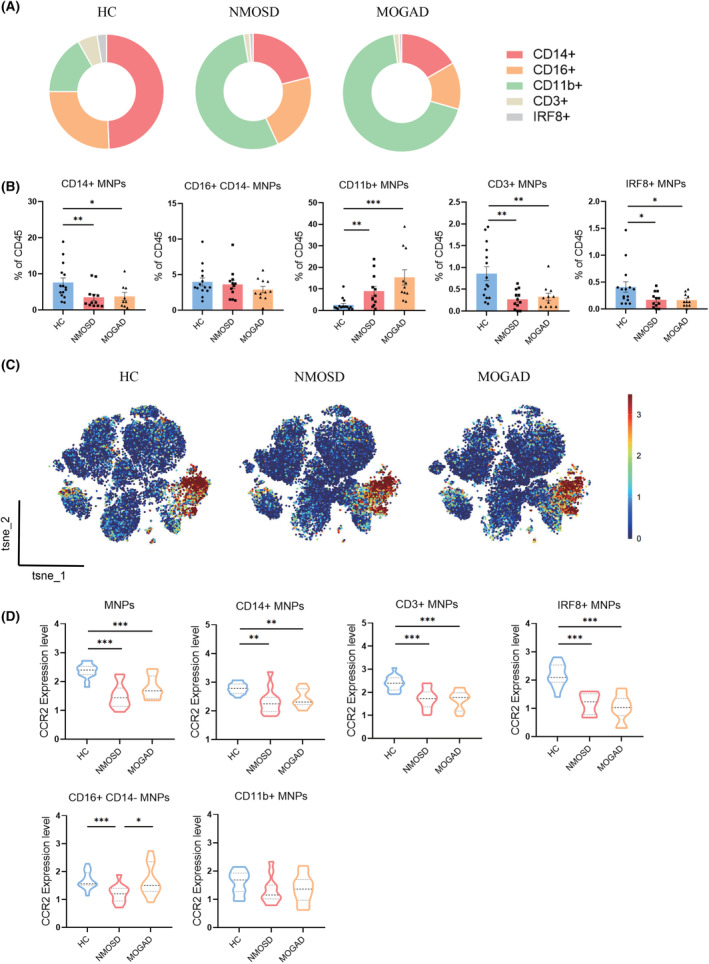
Abundances of MNPs subsets in patients with AQP4‐IgG seropositive NMOSD or MOGAD compared to HC. (A) Ring charts showed the relative frequencies of different MNPs subsets. (B) The column graphs showed mean frequencies of MNPs subset as % of total CD45+ cells (HC = 15, NMOSD = 12, MOGAD = 11). (C) t‐SNE maps showed the distribution of CCR2+ MNPs in HC and patients with AQP4‐IgG seropositive NMOSD or MOGAD groups. (D) Violin plots showed the volume of all samples from each group by width and lines correspond to the 25th percentile, median, and 75th percentile. Data were presented as mean ± SEM; bars represent maximum and minimum values, and dots represent individual samples. *p < 0.05; **p < 0.01; ***p < 0.001.

Monocytes, macrophages, and DCs are considered MNPs, which play important roles in inflammatory disorders.^15^, ^16^ We observed a lower proportion of CD14+ MNPs (cluster 15) and a higher proportion of CD11b+ MNPs (cluster 4, 22) in patients with AQP4‐IgG seropositive NMOSD and MOGAD groups, compared to the HC group (Figure 2A,B). Compared to HC group, the box charts displayed a marked decrease of CD3+ MNPs (cluster 20) and IRF8+ MNPs (cluster 24) in both NMOSD and MOGAD patients. No significant change in the proportion of CD16 + CD14‐MNPs (cluster 1, 23; Figure 2B).

Previous studies reported that exposure of NMOSD‐targeted cell astrocytes to NMO‐IgG would induce chemoattractant chemokine ligand 2 (CCL2) expression to increase or release.^14^, ^17^ As we know, CCL2 binds to its receptor, CCR2, which is primarily produced in activated MNPs and NK cells.^18^, ^19^ For MNPs, after attaching to the chemokine CCL2, they migrate out of the bone marrow via the critical receptor CCR2. t‐SNE maps display CCR2+ MNPs percentage and distribution of NMOSD, MOGAD, and HC (Figure 2C). Compared to HC, violin plots show CCR2‐expressing MNPs reduction in NMOSD and MOGAD patients (Figure 2D). Also, in both NMOSD and MOGAD patients, CCR2‐expressing CD14+ MNPs, CD3+ MNPs, and IRF8+ MNPs decreased markedly. Compared to HC group and MOGAD patients, CCR2‐expressing CD16+ CD14‐MNPs were lower in NMOSD patients. No significant changes were discovered in CCR2‐expressing CD11b+ MNPs among NMOSD, MOGAD, and HC groups (Figure 2D).

### 3.3 CCR2 gene expression was elevated significantly in both AQP4‐IgG seropositive NMOSD and MOGAD PBMCs samples

**FIGURE 3:**
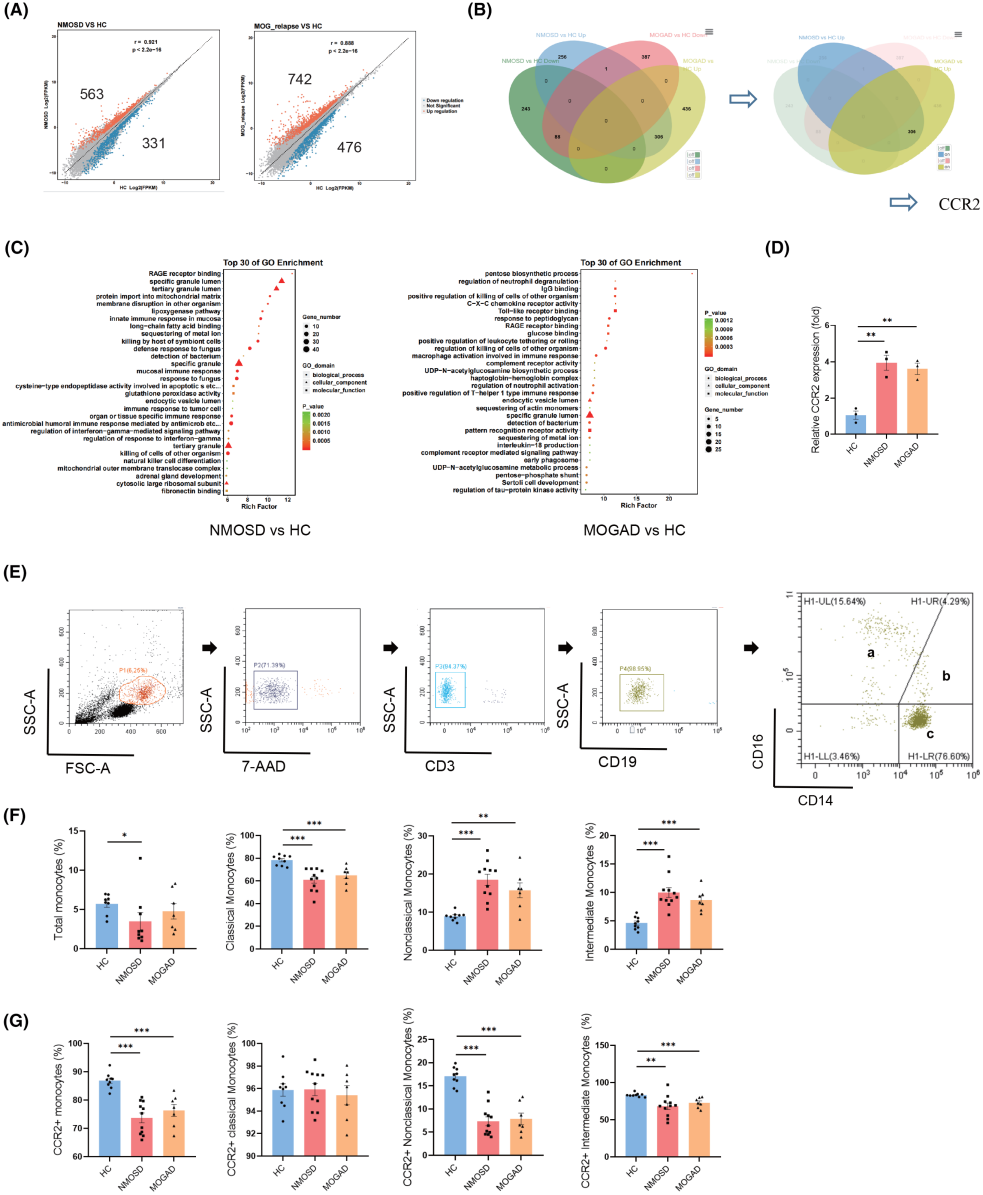
Identification subsets of MNPs according to CCR2 gene screening from RNA sequencing datasets. (A) Scatter plot showed UP or DOWN DEGs between peripheral blood of AQP4‐IgG seropositive NMOSD or MOGAD patients versus HC group. (Log [Fold change] > 1.5, *p* < 0.05) (B) Venn diagram showed the overlap of UP or DOWN DEGs in PBMCs from patients versus HC group. (https://jvenn.toulouse.inrae.fr/app/example.html) (C) Top 30 pathways of UP genes by GO enrichment analysis in NMOSD (left) and MOGAD (right). (D) Identification of the CCR2 expressions on PBMCs increasing in AQP4‐IgG seropositive NMOSD and MOGAD patients by qPCR analysis (*n* = 3). (E) The gating strategy in fluorescence cytometry. a: CD14‐CD16++ monocytes (nonclassical), b: CD14++CD16+ monocytes (intermediate), c: CD14++CD16‐ monocytes (classical). (F) The proportion of total monocytes or classical, nonclassical and intermediate three subsets (HC = 9, NMOSD = 11, MOGAD = 7). (G) The column graphs showed the percentage of CCR2 positive monocytes of total monocytes in PBMCs and three subsets in monocytes, respectively. Bar charts show the percentage of monocytes and each subpopulation (HC = 9, NMOSD = 11, MOGAD = 7). Percentage data were presented as mean ± SEM; bars represent maximum and minimum values, and dots represent individual samples. *p < 0.05; **p < 0.01; ***p < 0.001.

We used scatter to identify key genes in datasets with DOWN or UP differentially expressed genes (DEGs). In NMOSD, there were 331 DOWN and 563 UP DEGs as compared to the HC group. MOGAD patients had 476 DOWN and 742 UP DEGs. The distribution of fold changes in DEGs determined from RNA sequencing of PBMCs shows comparable tendencies in NMOSD and MOGAD patients (Figure 3A). Applying Venn diagram software, we discovered one of the upregulated genes that blood samples from patients with NMOSD and MOGAD had considerably higher CCR2 expression than HC (Figure 3B). Interestingly, monocyte extravasation, DC chemotaxis, and the inflammatory response to wounding pathways were considerably enriched in the upregulated genes CCR2 (Figure 3C). The CCR2 elevation in patients was further validated by qPCR analysis (Figure 3D).

### 3.4 Monocytes, especially CCR2+ monocytes decreased significantly in patients validated by fluorescence cytometry

Monocytes are derived from innate blood cells and are classified into three subsets based on their expressions of CD16 and CD14 surface antigens: classical (CD16−CD14++), nonclassical (CD16+CD14−), and intermediate (CD16+CD14++).^20^ The percentage of total monocytes and its 3 subsets were identified; furthermore, the frequencies of CCR2+ monocytes and each subset were also measured in HC, AQP4‐IgG seropositive NMOSD, and MOGAD patients by spectral flow cytometry (Figure 3E). Comparing NMOSD patients to HC, the amount of total monocytes was considerably lower, mainly decreased in the subset of classical monocytes, while nonclassical and intermediate monocytes showed a significant rise (Figure 3F). Consistently, the CCR2+ monocytes, CCR2+ nonclassical monocytes, and CCR2+ intermediate monocytes notably decreased in both patients. Regarding CCR2+ classical monocytes, there were no appreciable variations between the NMOSD, MOGAD, and HC groups (Figure 3G).

### 3.5 T‐cell subsets profiles in AQP4‐IgG seropositive NMOSD and MOGAD patients

**FIGURE 4:**
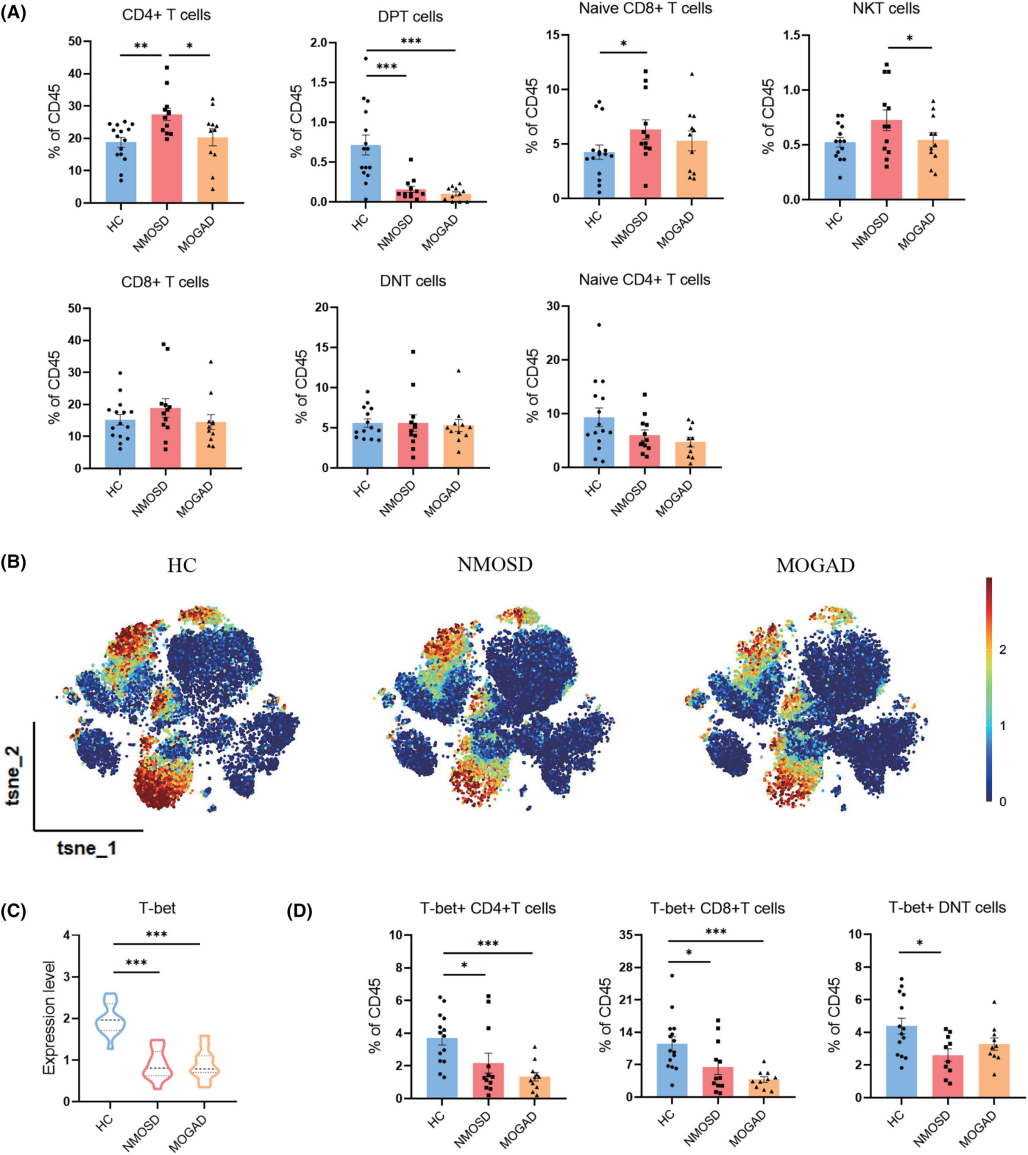
T lymphoid lineage profiles of patients with AQP4‐IgG seropositive NMOSD or MOGAD compared to HC analyzed by CyTOF. (A) Changes in several T‐cell subsets of NMOSD and MOGAD patients compared to HC (HC = 15, NMOSD = 12, MOGAD = 11). (B) Statistical t‐SNE map showed different T‐bet abundance using phenograph analysis. (C) Violin plot showed the T‐bet expression of AQP4‐IgG seropositive NMOSD and MOGAD decreasing significantly compared to HC. (D) The column graphs showed mean frequencies of each subset as % of total T cells (HC = 15, NMOSD = 12, MOGAD = 11). Data were presented as mean ± SEM; bars represent maximum and minimum values, and dots represent individual samples of each group. *p < 0.05; **p < 0.01; ***p < 0.001.

The fraction of CD4+ T cells (cluster 3, 5, 6, 17, 21) in NMOSD patients increased significantly, compared to both HC and MOGAD groups. Compared to HC group, the box charts displayed marked decrease in CD4 + CD8+ double‐positive T cells (DPT; cluster 14) in both NMOSD and MOGAD patients. NMOSD patients had a larger fraction of naïve CD8+ T lymphocytes (cluster 7). Between MOGAD and NMOSD, there was a substantial difference in the frequency of NKT cells (cluster 18). No significant alteration among the percentage of CD4‐CD8‐double‐negative T cells (DNT; cluster 12, 19), CD8+ T cells (cluster 2, 7, 10, 11), and naïve CD4+ T cells (cluster 3; Figure 4A).

As we know, T‐bet has been recognized as a crucial transcription factor associated with cell‐mediated immunity that helps naïve T cells differentiate into the Th1 subgroup.^21^, ^22^ In present study, analysis of the T‐bet+ T‐cell fraction was done. The ViSNE map displayed the distributions of T‐bet+ T cells in HC, NMOSD, and MOGAD patients. (Figure 4B). The violin plot displayed that the T‐bet expression on T cells of NMOSD and MOGAD patients was substantially decreased compared to HC (Figure 4C). In NMOSD and MOGAD patients compared to HC, there were significant drop in both clusters, 17 (T‐bet+CD4+) and 2, 11 (T‐bet+CD8+; Figure 4D). Only NMOSD patients had a significant decrease in cluster 19, which is defined by T‐bet+CD4‐CD8‐DNT cells; patients with MOGAD exhibited a tendency toward decline (Figure 4D).

### 3.6 CD56+ NK cells and CD20+ B cells decreased in both patients with NMOSD and MOGAD, proportion of CD38+CD138+ B cells may differentiate MOGAD from NMOSD

**FIGURE 5:**
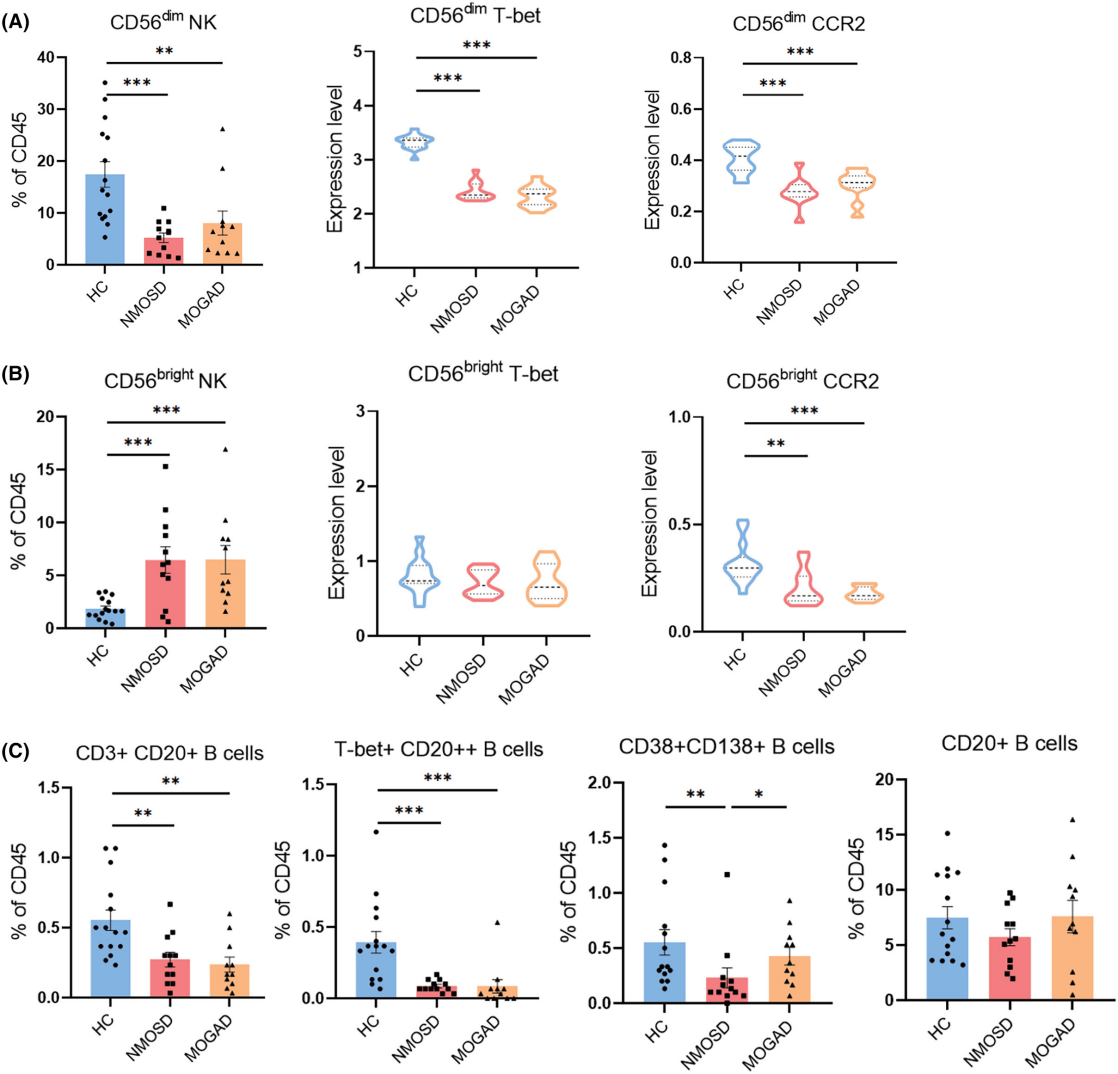
Percentages of NK and B cells subsets in patients with AQP4‐IgG seropositive NMOSD or MOGAD compared to HC. (A) Percentage of CD56^dim^ NK cells in PBMCs samples (left). Violin plots showed the distribution of CD56^dim^ T‐bet (middle) and CD56^dim^ CCR2 (right) subsets, respectively. (B) Percentage of CD56^bright^ NK cells in PBMCs samples (left). Violin plots show the distribution of CD56^bright^ T‐bet (middle) and CD56^bright^ CCR2 (right) subsets, respectively. (C) Percentage of CD3 + CD20+, T‐bet+CD20++, CD38 + CD138+ B cells and CD20+ B cells in PBMCs samples (HC = 15, NMOSD = 12, MOGAD = 11). Percentage data were presented as mean ± SEM; bars represent maximum and minimum values, and dots represent individual samples. *p < 0.05; **p < 0.01; ***p < 0.001.

Human NK cells account for about 15% of all circulating lymphocytes.^23^ Based on CD56 and CD16 surface markers relative expression,^24^ two major subsets of CD56^dim^ (CD16+) and CD56^bright^ (CD16‐) are subdivided. Compared to HC, a significant decrease of CD56^dim^ NK cells (cluster 16) in NMOSD and MOGAD. T‐bet and CCR2 expressing CD56^dim^ NK cells were also reduced markedly in NMOSD and MOGAD patients (Figure 5A). On the contrary, the amount of CD56^bright^ NK cells (cluster 13) was considerably increased in NMOSD and MOGAD. No significant differences in T‐bet expressing‐CD56^bright^ NK cells were discovered among the 3 groups. Compared to HC group, CCR2 expressing‐CD56^bright^ NK cells were considerably decreased in patients (Figure 5B).

As AQP4‐IgG seropositive NMOSD and MOGAD are both autoantibodies mediated diseases, B cells are important in pathogenesis. As B cells mature, plasma cells are produced. These cells provide antigens to stimulate the growth and activation of autoimmune T cells and produce pathogenic IgG.^25^, ^26^ By CyTOF, we identified four clusters of B cells based on the patterns of expression markers: cluster 26 (CD3+CD20+ B cells), cluster 27 (T‐bet+CD20++ B cells), cluster 8 (CD38+CD138+ B cells) and cluster 9 (CD20+ B cells). Cluster 26 and 27 decreased significantly in both patient groups compared to HC group; however, compared to HC groups and MOGAD patients, the box charts showed a marked decrease of CD38+CD138+ B cells (cluster 8) in NMOSD patients. No appreciable differences were discovered in CD20+ B cells among NMOSD, MOGAD, and HC groups (Figure 5C).

### 3.7 Comparison of PBMCs specific phenotypes between NMOSD patients during acute and remission phases

**FIGURE 6:**
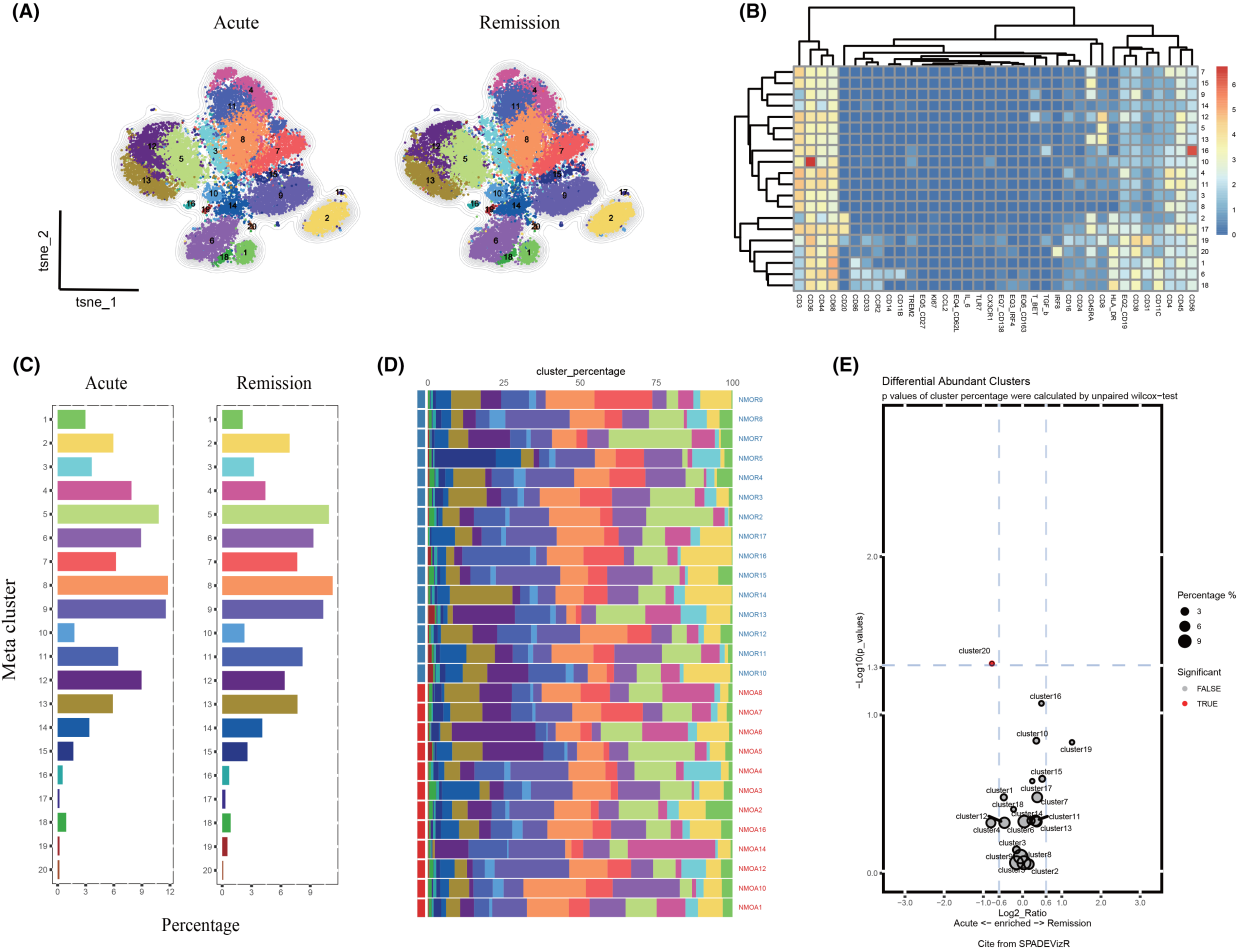
Percentages of PBMCs phenotypes in patients with AQP4‐IgG seropositive NMOSD during acute (*n* = 12) and remission (*n* = 15) phases. (A) According to the relative expression markers, high dimensional mass cytometry clustering revealed distinct cell type subpopulations. CD45+ PBMCs were divided into 20 clusters. (B) A heatmap showed the relative expression levels of selected markers in the 20 t‐SNE clusters. (C) Cluster abundances in patients with AQP4‐IgG seropositive NMOSD during acute and remission phases. (D) A histogram showed the cluster percentage of all participants in the 20 t‐SNE clusters. (E) The SPADEVizR method was used to analyze differences in immune composition between acute and remission, and the results were presented on a volcanic map. (NMOA: patients with NMOSD during acute phases; NMOR: patients with NMOSD during remission phases).

PBMCs samples from 12 and 15 NMOSD patients during acute and remission phases, respectively, were collected for phenotypes identification. ViSNE map displayed a similar distribution of PBMCs phenotypes between acute and remission (Figure 6A). Heatmap showed the expression profiles from each cluster (Figure 6B). The percentage of every cluster was displayed in Figure 6C. A histogram was used to show each participant's cluster proportion. (Figure 6D). Although similar changes were observed between acute and remission in NMOSD patients, a significantly higher abundance of IRF8+ cells were found during acute phase (cluster 20; Figure 6E).

## 4 DISCUSSION

Overlap in clinical, demographic, and radiographic characteristics of AQP4‐IgG seropositive NMOSD and MOGAD confuse or hinder neurologists from making correct decisions at the onset for therapeutic strategies. Deeply and comprehensively understanding the profiles of PBMCs in patients will benefit disease treatment and novel drug development. Here, we used CyTOF in conjunction with phenograph‐based algorithms to deliver a summary of the environment of circulating immune cells in AQP4‐IgG seropositive NMOSD or MOGAD during acute stage before drug treatments compared to HCs. By employing CyTOF analysis on a collection of 36 markers that are either intrinsic or expressed on PBMCs, we were able to distinguish distinct populations of MNPs, T, NK, and B cells for the purpose of distinguishing patients from healthy individuals. Further RNA sequencing, qPCR quantification, and spectral flow cytometry further identified our findings in MNPs. Our data offer the opportunity to discriminate or diagnose these two similar diseases early based on PBMCs characteristics.

Using a combination of clustering methods, we found five MNPs populations in the blood of healthy persons and those suffering from NMOSD or MOGAD (Figure 2). We observed a considerable decrease in the percentage of CD14+ MNPs and an increase in the fraction of CD11b + MNPs in demyelinated patients. However, there is no change in MNPs defined by CD16 in patients. In addition to CD16, CD14, CD11b, CD11c, IRF8, IRF4, and CCR2 are the most significant markers for defining MNP subsets.^27^ CCR2 expression rise was detected using RNA sequencing and qPCR in all cases. We additionally confirmed the percentage of CCR2+ monocyte subgroups in another cohort (Figure 3). It has been demonstrated that nonclassical monocytes patrol blood vessels, protect the vascular endothelium, preserve vascular homeostasis, and remove dying endothelial cells.^20^ We discovered a considerable decrease of CCR2+ nonclassical monocytes in both patients (Figure 3G), with an average of about 7.35% and 7.86% for NMOSD and MOGAD, respectively, that can potentially be involved in the pathogenesis of disease. Previous studies proved that NMO‐IgG stimulated an immunological response, including CCL2 release in rat astrocyte cultures.^14^, ^28^ Considering that CCL2 is a strong chemotactic stimulant of CCR2+ myeloid cells and is essential for the creation of inflammatory infiltration in the brain, there were much lower CCR2+ cells in the periphery of NMOSD patients.

Autoantibodies are hallmarks of NMOSD and MOGAD. MOGAD is not synonymous with AQP4‐IgG seronegative NMOSD.^29^ According to retrospective research, therapeutic techniques targeting CD20+ B cells function effectively in NMOSD but not in MOGAD.^30^ To describe the diversity of CD3+ T cells responses to pathogenesis driven by autoantibodies, populations of CD4 and CD8 T cells, DNT and DPT cells, NKT cells, and naïve T cells were further analyzed (Figure 4). We observed that individuals with NMOSD had considerably higher proportions of naïve CD8+ T and CD4+ T cells as compared to those in the HC group, but DPT cells were generally lower in NMOSD and MOGAD. Between NMOSD and MOGAD, the frequencies of CD4+ T and NKT cells were considerably different. While CD8+ T cells predominate in multiple sclerosis (MS), CD4+ T cells are more numerous when perivascularly infiltrated in the brain parenchyma lesions with NMOSD and MOGAD.^31^ The peripheral T cells recognized the AQP4 peptides, oriented to develop into helper T‐cell subtype 17 (Th17), and helped B cells to undergo plasmablast differentiation into AQP4‐IgG‐releasing plasmablasts.^32^ DPT cells declined simultaneously in NMOSD and MOGAD, despite the fact that these conditions are today acknowledged as distinct disease entities.

Abnormal T‐cell activity is associated with autoimmune and inflammatory diseases, and distinct T‐cell subsets may give resistance to distinct types of infections.^22^ Th1‐specific T‐box transcription factor T‐bet is expressed in NK and Th1 cells and regulates the expression of IFN‐γ, a hallmark Th1 cytokine.^21^ T‐bet positive T cells or their CD4+ and CD8+ subgroups decreased in MOGAD and NMOSD (Figure 4C,D). The peripheral blood becomes inflamed due to T cells or their subsets malfunctioning, which permits the formation of autoantibodies and their arrival into the CNS, resulting in a devastating onslaught.^33^ The percentages of T‐bet‐expressing DNT cells in NMOSD were considerably reduced than in the HC (Figure 4D), indicating extensive T‐cell dysfunction in NMOSD.

Inflammation and immunological surveillance are critical functions of cytotoxic immune cells. NK or NKT cells and CD8+ T cells are the two different subtypes of cytotoxic cells. NK cells were considered innate immunity cells due to their significant effector functions in anti‐tumoral immune responses and strong cytotoxicity against virus‐infected cells.^34^ As CCR2 and T‐bet were both expressed on NK cells, We also assessed the percentage of T‐bet or CCR2 expressions in two NK subgroups, CD56^bright^ and CD56^dim^ NK cells (Figure 5). T‐bet expression only reduced in CD56^dim^ NK cells; however, CCR2 expression dramatically decreased on both CD56^bright^ and CD56^dim^ NK cells. This suggests that both CD56^bright^ and CD56^dim^ subsets of immune surveillance and cytotoxicity are dysfunctional.

CD3+CD20+ B cells are a population of B cells co‐expressing CD3 molecularly in T cells that make up to 0.55% of the peripheral blood (Figure 5C). Recent research has demonstrated that CD3+CD20+ B cells exhibit harmful activity in autoimmune disorders, and patients may benefit from immunotherapy that targets CD20. Meanwhile, CD3+CD20+ ovarian cancer and HIV infection may benefit from the great propensity of B cells to produce IFN‐γ.^35^ Despite total B‐cell depletion, patients with MOGAD treated with rituximab had relapsed and seemed less robust than those with NMOSD. However, a considerable number of MOGAD patients may experience severe infections and hypogammaglobulinemia as a result of rituximab‐induced CD3+CD20+ B‐cell depletion.^34^ Low levels of CD3+CD20+ and T‐bet+CD20++ B cells in patients may be due to disease modification treatments previously. CD38+CD138+ B cells have been characterized as long‐lived plasma cells (LLPCs), which sustained responses to viral infections for historical exposure.^36^ Higher levels of CD38+CD138+ B cells in MOGAD patients than in NMOSD may represent an explanation for the bad prognosis of MOGAD after rituximab treatment. In addition, similar changes were observed between acute and remission in NMOSD patients; a significantly higher abundance of IRF8+ cells was found during the acute phase (Figure 6). IRF8 has been associated with an increased risk of MS, according to recent epidemiological research.^37^

Actually, there are several limitations to this study. First, the study may have selection bias because of the limited sample size and the fact that all of the patients came from a single center. Second, our research did not use in vitro or in vivo functional studies to confirm the functionality of the immune cells. Third, the heterogeneous characteristics of B‐cell populations are indicated by the fact that serum AQP4‐IgG titers do not necessarily correlate with B‐cell depletion in patients with NMOSD. In the current work, we grouped B cells using standard markers like CD20 or CD19‐specific antibody‐secreting cells (ASCs). Future research should focus on identifying other markers, including CD27, MS4A1, MK67, and SDC1.^38^ Then, comorbidities such as diabetes, hypercholesterolemia, and hypertension may influence PBMCs constitution. Few comorbidities may be present in any of the healthy control patients who are enrolled in the present investigation.

## 5 CONCLUSIONS

Together, our findings provided a thorough description of the circulating PBMC landscape for AQP4‐IgG seropositive NMOSD and MOGAD revealed the profiles of peripheral blood immune cells, which may play potential roles in disease processing or drug treatment responses. Our results also provided evidence for the interpretation of different treatment responses or clinical features of MOGAD and AQP4‐IgG seropositive NMOSD and gave a hint at promising directions for precision therapy.

## AUTHOR CONTRIBUTIONS

Conceptualization, L.Y. and J.L.; methodology, M.Y. and W.W.; validation, L.Y. and J.L.; formal analysis, M.Y., W.W., J.S., T.G., J.B., F.X., Y.L., H.C., Y.W. and X.Z.; investigation – tissue preparation, J.S., T.G. and J.B.; investigation – experiments and data acquisition, M.Y., W.W., J.S. and T.G.; investigation – patient data, M.Y., J.S., T.G., Y.L., H.C., Y.W. and X.Z.; writing – original draft, M.Y. and L.Y.; supervision, L.Y. and J.L.

## FUNDING INFORMATION

This work was supported by Beijing Natural Science Foundation (7212030, 7212029), National Key Research and Development Program of China (No. 2019YFC0121202), and National Natural Science Foundation of China (No. 82271374).

## CONFLICT OF INTEREST STATEMENT

No, there is no conflict of interest.

## CONSENT FOR PUBLICATION

Not applicable.

## Supporting information


Figure S1.

Table S1.

Table S2.

Table S3.

Table S4.

Table S5.

## Data Availability

The original data are available and the raw CyTOF data used and analyzed in the current study are available from the corresponding author upon reasonable request.
