# Supplementary figures and images for: The landscape of PBMCs in AQP4‐IgG seropositive NMOSD and MOGAD, assessed by high dimensional mass cytometry
Source: CNS Neurosci Ther. 2024 Feb 9;30(2):e14608. doi: 10.1111/cns.14608 (PMC10853888; doi:10.1111/cns.14608)

**A**

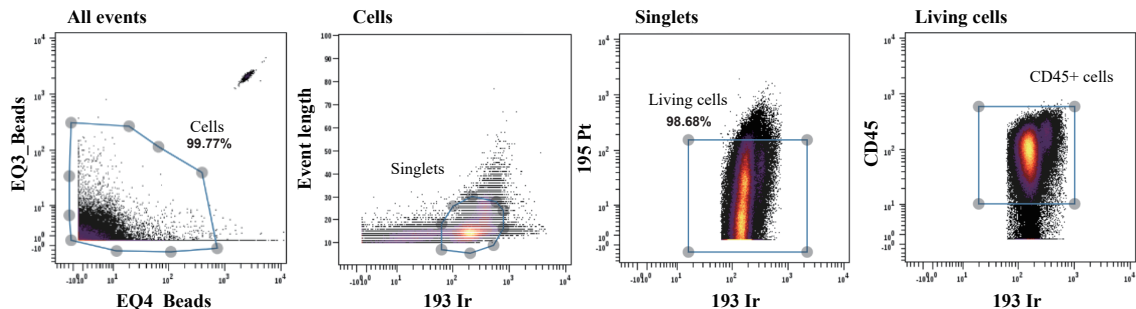

**B**

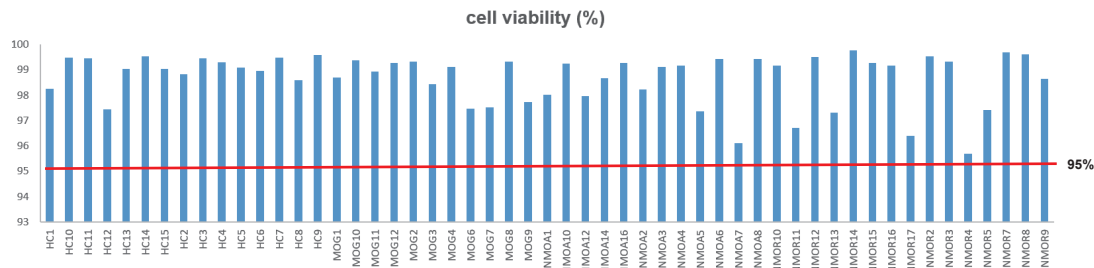

Supplement: Supplementary file 1 — Figure S1. [file CNS-30-e14608-s003.pdf]
