# Supplementary material for: The landscape of PBMCs in AQP4‐IgG seropositive NMOSD and MOGAD, assessed by high dimensional mass cytometry
Source: CNS Neurosci Ther. 2024 Feb 9;30(2):e14608. doi: 10.1111/cns.14608 (PMC10853888; doi:10.1111/cns.14608)
Supplement: Supplementary file 2 — Table S1. [file CNS-30-e14608-s005.docx]

**Supplementary Table 1 Detailed demographic data and clinical characteristics of** **patients who participated in mass cytometry**

| **No.** | **Sex** | **Age**  **(year)** | **EDSS** | **Serum AQP4 antibody titer (1:)** | **Serum MOG antibody titer (1:)** | **Disease classification** | **Lesion region** | **Treatments^a^** | **Rituximab infusion last time^b^** | **Cardinal symptom** | **Previous experience of the drug** |
| --- | --- | --- | --- | --- | --- | --- | --- | --- | --- | --- | --- |
| NMOA1 | F | 30 | 5 | 32 | - | myelitis | T1-8 | methylprednisolone/  mycophenolate mofetil | - | limb weakness/algesia/bowel and bladder dysfunction | Sep 11, 2019: steroid pulse therapy + prednisone |
| NMOA2 | F | 44 | 2.5 | 32 | - | myelitis | C3-4 | methylprednisolone | - | numbness and weakness of limbs/algesia | - |
| NMOA3 | M | 72 | 6 | 100 | - | myelitis | C1-3 T11-12 | methylprednisolone | - | numbness and weakness of limbslimb weakness/bowel and bladder dysfunction | - |
| NMOA4 | M | 37 | 4 | 10 | - | brainstem encephalitis | right pontine | prednisone | - | blurred vision | - |
| NMOA5 | F | 50 | 8 | 32 | - | myelitis | C3-4 | methylprednisolone/tocilizumab | Jul 11, 2018 | numbness and weakness of limbs | May 2003: steroid; Nov 2003: interferon; Mar 2015: steroid + interferon; Apr 2015: interferon + MMF + IVIG; Aug 2015: MP + CyA + RTX; Jul 11, 2018: RTX; May 30,2019/Nov 18 2019: MP + tacrolimus |
| NMOA6 | M | 34 | 4.5 | 10 | - | myelitis/optic neuritis | Ⅱ C5 | methylprednisolone/plasma adsorption | - | numbness and weakness of limbs/blurred vision | - |
| NMOA7 | F | 30 | 4 | 100 | - | myelitis/optic neuritis | T4-8 | methylprednisolone | Dec 20, 2019 | limb weakness/algesia | Apr 20, 2019: DXM + IVIG; Jun 4, 2019: prednisone + RTX; Jul 1, 2019/Dec 20, 2019: RTX |
| NMOA8 | F | 44 | 6 | 32 | - | myelitis | C6-T2 | methylprednisolone | - | numbness and weakness of limbs | - |
| NMOA10 | F | 43 | 2.5 | 32 | - | brainstem encephalitis | C2 T4 T6-7 medulla | methylprednisolone | - | nausea and vomiting/blurred vision/walk unsteadily | - |
| NMOA12 | F | 46 | 4 | 10 | - | myelitis | C3-4 | methylprednisolone/mycophenolate mofetil | - | numbness and weakness of limbs | - |
| NMOA14 | F | 74 | 3 | 320 | - | myelitis | T3-4 T11 | methylprednisolone | - | numbness and weakness of limbs | - |
| NMOA16 | F | 52 | 7 | 320 | - | myelitis | C3-T7 medulla | methylprednisolone/gamma globulin | - | numbness and weakness of limbs | Sep 11, 2019: steroid pulse therapy; Oct 31, 2019: CTX + prednisone |
| NMOR2 | F | 55 | 3 | 10 | - | myelitis | T3-5 | rituximab | Nov 27, 2020 | - | Jun 2017/Dec 2017: MP; Jun 2018: prednisone + tocilizumab; Oct 2020: MP + prednisone + RTX; Nov 27, 2020: RTX |
| NMOR3 | F | 19 | 2 | 100 | - | myelitis | C3 | rituximab | Mar 24, 2021 | - | Feb 1, 2021: MP + IVIG; Mar 10,2021/Mar 24, 2021: RTX |
| NMOR4 | F | 35 | 4 | 320 | - | myelitis | C1 C3 T1-8 | rituximab | Aug 12, 2020 | - | Aug 2017: steroid; Sep 2019: MP + prednisone; Jun 2020/Feb 2020/Mar 2020/Aug 2020: RTX |
| NMOR5 | F | 30 | 2.5 | 32 | - | myelitis | C7-T4 | rituximab | Mar 29, 2021 | - | Dec 7, 2016: MP + AZA; Mar 5, 2019: MP + IVIG; Apr 2019/Oct 2019: RTX; Aug 6, 2020: steroid; Mar 29,2021: RTX |
| NMOR7 | F | 65 | 1 | 320 | - | optic neuritis | T2-3 | rituximab | Nov 14, 2020 | - | Dec 3,2017: steroid + prednisone + IVIG; Oct 14, 2019: steroid + RTX; Nov 22, 2019/Nov 14, 2020: RTX |
| NMOR8 | F | 46 | 2 | 10 | - | myelitis | C3-4 | - | - | - | Jun 25,2021: MP |
| NMOR9 | F | 43 | 2.5 | 32 | - | brainstem encephalitis | medulla C2 T3-4 | mycophenolate mofetil | - | - | Sep 15, 2020: steroid + prednisone |
| NMOR10 | F | 42 | 4.5 | 320 | - | myelitis | - | mycophenolate mofetil | - | - | Oct 2020/Nov 2021: steroid + prednisone + IVIG; |
| NMOR11 | F | 52 | 4 | 320 | - | myelitis | C3-4 T2-4 | rituximab | Sep 27, 2021 | - | Sep 11, 2019: steroid + prednisone; Sep 29,2019: CTX + prednisone; Sep 2021: steroid + IVIG + RTX |
| NMOR12 | F | 31 | 1 | 32 | - | brainstem encephalitis | Ⅱ medulla | rituximab | - | - | Jun 2014/Dec 2015: steroid; Jun 2016: prednisone;2017: CyA; Mar 2018: steroid; Aug 2018: CyA |
| NMOR13 | F | 44 | 1 | 32 | - | optic neuritis | C4-6 | rituximab | Mar 31, 2021 | - | Feb 2021: steroid; Mar 31, 2021: RTX |
| NMOR14 | F | 15 | 0 | 10 | - | brainstem encephalitis | - | - | - | - | - |
| NMOR15 | F | 59 | 6 | 320 | - | myelitis | T1-5 | rituximab | Oct 13, 2021 | - | Jun 25, 2019: steroid + RTX; Feb 2020/Nov 2020: RTX; Aug 29,2021: IVIG; Oct 13, 2021: RTX |
| NMOR16 | F | 49 | 4 | 320 | - | myelitis/optic neuritis | Ⅱ | rituximab | - | - | Aug 2021: steroid |
| NMOR17 | F | 52 | 7 | 32 | - | myelitis/optic neuritis | T3-5 | tocilizumab | - | - | May 19, 2021: steroid; Jun 8, 2021: tocilizumab |
| MOG1 | M | 37 | 0 | - | 10 | optic neuritis |  | methylprednisolone | - | left eye vision loss | May 26, 2021: MPSS + Prednisone |
| MOG2 | M | 78 | 3 | - | 10 | encephalitis/myelitis |  | gamma globulin | - | epilepsy/weakness of limbs | - |
| MOG3 | M | 37 | 4.5 | - | 10 | brainstem encephalitis |  | methylprednisolone | - | see things in pairs/eye movement disorder | - |
| MOG4 | M | 35 | 4 | - | 10 | encephalitis |  | gamma globulin | - | epilepsy | Feb 2, 2022: IVIG |
| MOG6 | F | 27 | 0 | - | 32 | encephalitis |  | gamma globulin | - | epilepsy | - |
| MOG7 | F | 20 | - | - | 32 | encephalitis |  | gamma globulin | - | epilepsy | - |
| MOG8 | F | 41 | - | - | 32 | optic neuritis |  | methylprednisolone | - | decreased vision in both eyes | Nov 1, 2021: steroid pulse therapy + Prednisone |
| MOG9 | F | 15 | 5 | - | 100 | encephalitis |  | methylprednisolone/gamma globulin | - | epilepsy | - |
| MOG10 | M | 43 | 4.5 | - | 100 | myelitis |  | methylprednisolone | - | double lower limbs weakness | 2014/2015/2016: IVIG + DXM |
| MOG11 | M | 18 | 0 | - | 100 | encephalitis |  | gamma globulin | - | epilepsy | - |
| MOG12 | M | 34 | 2 | - | 10 | optic neuritis |  | methylprednisolone | May 18, 2021 | right eye vision loss | Mar 5, 2020: citicoline; May 2, 2020: MP + IVIG + RTX; Jun 2020/Dec 2020/May 2021: RTX |

Underline: patients who participated in RNA-seq; NMOA: patients with NMOSD during acute phases; NMOR: patients with NMOSD during remission phases; ^a^: Samples were collected before treatments; ^b^: Samples from remission were collected without treatment or before rituximab infusion; -: Patients were antibody negative or did not receive medication; MPSS: methylprednisolone sodium succinate; IVIG: intravenous gamma globulin; DXM: dexamethasone; MP: methylprednisolone; RTX: rituximab; CTX: cyclophosphamide; MMF: mycophenolate mofetil; CyA: cyclosporin; AZA: azathioprine.
