# Supplementary material for: The landscape of PBMCs in AQP4‐IgG seropositive NMOSD and MOGAD, assessed by high dimensional mass cytometry
Source: CNS Neurosci Ther. 2024 Feb 9;30(2):e14608. doi: 10.1111/cns.14608 (PMC10853888; doi:10.1111/cns.14608)
Supplement: Supplementary file 3 — Table S2. [file CNS-30-e14608-s004.docx]

**Supplementary Table 2 Mass cytometry panel**

| **Marker** | **Metal isotope** | **Source** | **Identifier** | **Description** |
| --- | --- | --- | --- | --- |
| CD45 | 89Y | Fluidigm | Cat#3089003B | Hematopoietic marker |
| TREM2 | 141Pr | R＆D | [MAB17291](https://www.rndsystems.com/cn/products/human-mouse-trem2-antibody-237920_mab17291) | Transmembrane receptor |
| CD19 | 142Ce | Biolegend | Cat#302247 | B cell |
| CD45RA | 143Nd | Fluidigm | Cat#3143006B | Naïve T cell |
| CD31 | 144Nd | Fluidigm | Cat#3144023B | Glycoprotein |
| CD16 | 145Nd | Fluidigm | Cat#3148004B | NK cell |
| TLR7 | 146Nd | R＆D | [MAB5875](https://www.rndsystems.com/cn/products/human-tlr7-antibody-533707_mab5875) | Transmembrane receptor |
| CD86 | 147Sm | abcam | ab242020 | Glycoprotein |
| CD4 | 148Sm | Fluidigm | Cat#3145001B | CD4 T cell |
| CD3 | 149Sm | Biolegend | Cat#300502 | T cell |
| CD11c | 150Sm | R＆D | [MAB1777](https://www.rndsystems.com/cn/products/human-cd11c-antibody-icrf-39_mab1777) | Glycoprotein |
| IRF4 | 151Eu | abcam | ab240071 | Transcription factor |
| CCL2 | 152Gd | Biolegend | 502601 | Chemokine |
| CD62L | 153Eu | Fluidigm | Cat#3153004B | Glycoprotein |
| IRF8 | 154Gd | abcam | ab306553 | Transcription factor |
| CD14 | 156Gd | Biolegend | Cat#301802 | Monocyte marker |
| CD33 | 158Gd | Biolegend | 303302 | Myeloid marker |
| CD44 | 159Tb | abcam | ab255946 | Glycoprotein |
| CD38 | 160Dy | R＆D | MAB2404 | Glycoprotein |
| T-bet | 161Dy | Biolegend | Cat#644802 | Transcription factor |
| CD8 | 162Dy | Fluidigm | Cat#3146001B | CD8 T cell |
| TGF-β | 163Dy | Fluidigm | Cat#3163010B | Cytokine |
| CD56 | 164Dy | Fluidigm | Cat#3155008B | NK cell |
| CD27 | 165Ho | R＆D | [AF382](https://www.rndsystems.com/cn/products/human-cd27-tnfrsf7-antibody_af382) | Glycoprotein |
| CCR2 | 166Er | R＆D | [MAB150](https://www.rndsystems.com/cn/products/human-ccr2-antibody-48607_mab150) | Chemokine receptor |
| CD20 | 167Er | R＆D | [MAB4225](https://www.rndsystems.com/cn/products/human-cd20-antibody-396444_mab4225) | B cell |
| Ki67 | 168Er | Fluidigm | Cat#3168001B | Proliferate antigen |
| CD24 | 169Tm | Fluidigm | Cat#3169004B | Glycoprotein |
| IL-6 | 170Yb | R＆D | [MAB2061](https://www.rndsystems.com/cn/products/human-il-6-antibody-1936_mab2061) | Cytokine |
| CX3CR1 | 171Yb | R＆D | AF5825 | Chemokine receptor |
| CD36 | 172Yb | R＆D | [MAB19551](https://www.rndsystems.com/cn/products/human-cd36-sr-b3-antibody-255606_mab19551) | Glycoprotein |
| CD68 | 173Yb | Fluidigm | Cat#3171011B | Glycoprotein |
| HLA-DR | 174Yb | Fluidigm | Cat#3174001B | MHC class II molecule |
| CD163 | 175Lu | R＆D | [MAB1607](https://www.rndsystems.com/cn/products/human-cd163-antibody-215927_mab1607) | Glycoprotein |
| CD138 | 176Lu | R＆D | [MAB2780](https://www.rndsystems.com/cn/products/human-syndecan-1-cd138-antibody-359103_mab2780) | Plasma cell marker |
| CD11b | 209Bi | Fluidigm | Cat#3209003B | Macrophage marker |
