# Supplementary material for: The landscape of PBMCs in AQP4‐IgG seropositive NMOSD and MOGAD, assessed by high dimensional mass cytometry
Source: CNS Neurosci Ther. 2024 Feb 9;30(2):e14608. doi: 10.1111/cns.14608 (PMC10853888; doi:10.1111/cns.14608)
Supplement: Supplementary file 4 — Table S3. [file CNS-30-e14608-s002.docx]

**Supplementary Table 3 Cell phenotype identification**

| **Immunocyte** | **Markers** |
| --- | --- |
| T cell | CD45+ CD3+ |
| CD4+ T cell | CD45+ CD3+ CD4+ |
| CD8+ T cell | CD45+ CD3+ CD8+ |
| Naïve T cell | CD45+ CD3+CD45RA+ |
| DNT cell | CD45+ CD3+CD4-CD8- |
| DPT cell | CD45+ CD3+CD4+CD8+ |
| NKT cell | CD45+ CD3+ CD56+ |
| Mononuclear phagocyte | CD45+CD3-CD19-CD68+ |
| Monocyte | CD45+ CD14+CD16+/- |
| Classcial monocyte | CD14++CD16- |
| Non-classcial monocyte | CD14-CD16++ |
| Intermediate monocyte | CD14+CD16+ |
| B cell | CD45+ CD19+/CD20+ |
| NK cell | CD45+ CD3- CD16+ CD56+ |
| CD56^bright^ NK cell | CD56^bright^CD16lo/− |
| CD56^dim^ NK cell | CD56^dim^CD16+ |
