# Supplementary material for: The landscape of PBMCs in AQP4‐IgG seropositive NMOSD and MOGAD, assessed by high dimensional mass cytometry
Source: CNS Neurosci Ther. 2024 Feb 9;30(2):e14608. doi: 10.1111/cns.14608 (PMC10853888; doi:10.1111/cns.14608)
Supplement: Supplementary file 5 — Table S4. [file CNS-30-e14608-s006.docx]

**Supplementary Table 4 The antibodies used in flow cytometric analysis**

| **Antibody** | **Information** |
| --- | --- |
| Alexa Fluor^®^ 700 anti-human CD3 Antibody | catalog no. 317340, BioLegend |
| PE anti-human CD19 Antibody | catalog no. 392506, BioLegend |
| Brilliant Violet 650^TM^ anti-human CD14 Antibody | catalog no. 301836, BioLegend |
| APC/Cyanine7 anti-human CD16 Antibody | catalog no. 302018, BioLegend |
| APC anti-human CD192 (CCR2) Antibody | catalog no. 357208, BioLegend |
| 7-AAD Viability Staining Solution | catalog no. 420403, BioLegend |
| Human TruStain FcX^TM^ | catalog no. 422302, BioLegend |
