# Supplementary material for: The landscape of PBMCs in AQP4‐IgG seropositive NMOSD and MOGAD, assessed by high dimensional mass cytometry
Source: CNS Neurosci Ther. 2024 Feb 9;30(2):e14608. doi: 10.1111/cns.14608 (PMC10853888; doi:10.1111/cns.14608)
Supplement: Supplementary file 6 — Table S5. [file CNS-30-e14608-s001.docx]

**Supplementary Table 5 Primers for qPCR**

| **Gene** | **Sense (5ˊ-3)** | **Anti-sense (5ˊ-3)** |
| --- | --- | --- |
| *CCR2* | CAGGTGACAGAGACTCTTGGGA | GGCAATCCTACAGCCAAGAGCT |
| *GAPDH* | GTCTCCTCTGACTTCAACAGCG | ACCACCCTGTTGCTGTAGCCAA |

All primers were designed and ordered from BGI Genomics Co., Ltd. Data were analyzed using the 2-^ΔΔ^Ct method.
